# Identification of multiomics map and key biomarkers in uveal melanoma with chromosome 3 loss

**DOI:** 10.1097/MS9.0000000000001585

**Published:** 2024-01-03

**Authors:** Xi Yong, Tengyao Kang, Tingting Li, Sixuan Li, Xuerui Hu, Xiang Yan, Fuzhao Zhang, Jianghua Zheng, Qin Yang

**Affiliations:** a Vascular Surgery Department of Affiliated Hospital of North Sichuan Medical College; b Hepatobiliary, Pancreatic and Intestinal Research Institute of North Sichuan Medical College; c Department of Clinical Medicine, North Sichuan Medical College; d Department of Pharmacy, The Second Affiliated Hospital of North Sichuan Medical College; e Infectious Diseases D of Affiliated Hospital of North Sichuan Medical College; f Endocrine Department of Affiliated Hospital of North Sichuan Medical College, Nanchong, Sichuan; g Department of Pharmacology, School of Pharmacy, Guangxi Medical University, Nanning, Guangxi, People’s Republic of China

**Keywords:** chromosome 3, GRIN2A, machine learning, multiomics, uveal melanoma

## Abstract

**Purpose:**

Chromosome 3 loss is an independent risk factor for uveal melanoma (UM), but its exact molecular mechanisms remain unclear. This study was designed to investigate the relationship between chromosome 3 loss and molecular alterations at multiple levels to construct a prognostic model.

**Methods:**

Forty-four UM cases with chromosome 3 loss (chr3 del group) and 36 UM cases without copy number variation on chromosome 3 (chr3 wt group) were collected from the Cancer Genome Atlas (TCGA). The TCGA dataset was subjected to a univariate Cox regression analysis to identify different expressed genes, and a subsequent random forest algorithm analysis revealed significant changes in different expressed genes, which were used to develop key biomarkers for UM. Following that, the immune cell infiltration analysis and drug sensitivity analyses were carried out. The UM cell line was then utilized to investigate the potential functions of the key biomarker via cell apoptosis, proliferation, cycle assays, WB, and RT-qPCR.

**Results:**

By analyzing the 80 cases data in TCGA, the authors unveiled molecular changes relevant to loss of chromosome 3 in UM as well as their poor survival. In addition, machine learning analysis identified three hub genes (GRIN2A, ACAN, and MMP9) as potential therapeutic targets. The differentially enriched pathways between the two groups were mainly about immune-system activity, and hub genes expression was also highly correlated with immune infiltration levels.

**Conclusion:**

Chromosome 3 loss has considerable clinical significance for UM, and GRIN2A may be useful in diagnosing, treating, and prognosticating the condition.

## Introduction

Highlights

- Exploring the molecular network that contributes to the loss of copy number of chromosome 3 and identifying potential mechanisms that underlie uveal melanoma metastasis.
- Key biomarkers include MMP9, GRIN2A, and ACAN.
- Infiltration levels of immune cells are highly correlated with the expression of hub genes.

Uveal melanoma (UM) is the most prevalent ocular melanoma in adults and has a poor prognosis due to its aggressive nature^1^. The tumor has a high probability of evading detection by the immune system and spreading to the liver. Enucleation was historically the most effective treatment for UM, but it is now more commonly treated with brachytherapy or proton beam irradiation. Metastasizing UM is a fatal for which there is no viable treatment^2^. Continued advances in the understanding of UM molecular mechanisms will facilitate the identification of prognostic markers and therapeutic targets^3^. Therefore, it is imperative that further research is conducted.

Chromosome copy number variants (CNVs) are associated with tumorigenesis due to their effects on gene function^4^. In UM, chromosome 3 loss can be an independent prognostic factor^5^. Patients with chromosome 3 loss often carry nonsynonymous mutations in BAP1, a tumor suppressor gene located on 3p21.1. In this case, the BAP1 mutant phenotype may result, and cells with this genotype are more advantageous under selection pressure from the immune-system and other factors, accelerating tumor progression^6,7^. Additionally, various molecular networks may contribute to the discovery of the mechanisms involved in the copy number loss on chromosome 3^8,9^. These networks comprise profiles of methylation, miRNA expression, and transcriptome expression. In this study, we utilized machine learning to construct an integrated molecular analysis to explore potential mechanisms influencing copy number loss on chromosome 3 and reveal potential key biomarkers from the TCGA-UM cohort with machine learning.

## Materials and methods

### Data processing and analysis

The TCGA database (https://portal.gdc.cancer.gov/) is the largest repository of cancer gene information. As raw SNP data in TCGA are not accessible to the public, utilize TCGAbiolinks^10^ to download mutation data, expression data, copy number data, and methylation data for 80 cases of TCGA-UM cases. To obtain copy number variation data at the chromosome level, SNP6 microarray data was analyzed with GISTIC2^11^ with default parameters. A total of 80 cases were categorized based on whether or not copy number loss on chr 3 occurred. A survival package was used to examine survival differences between groups of UM patients classified into chr3 Del and chr3 WT (44 vs 36). Using the maftools package^12^, nonsynonymous mutations were examined in the different groups.

### Differential expression analysis

To identify genes that were differentially expressed between the chr3 Del and chr3 WT groups, DESeq2^13^ was used to analyze mRNA and miRNA counts values. A differential methylation analysis was performed using the minfi package^14^ to identify probes with differential methylation levels, followed by pathway enrichment with the missMethyl package^15^.

### PPI network construction

STRING online database 11.0 (https://string-db.org)^16^ was used to explore the PPI network of modular genes. Using the Cytoscape software, the generated PPI networks were further visualized.

### Random survival forest models construction

Random survival forest^17^ is a machine learning method for processing survival data that is based on random forest, and the tree building rules are similar to those of random forest. First, a single-factor cox-risk proportional regression model was used to initially screen genes associated with prognosis in the training set, and randomly chosen training samples were used to build trees by extracting bootstrap samples, resulting in the construction of 1000 classification trees. Following the tree building process, several predictor variables were selected at random at each node for classification based on survival criteria containing survival time and truncated tail information. An exponential ranking or gene occurrence frequency was used to select genes from among the variables entering the model. Random survival forest decision trees are dichotomous survival trees that prevent the overfitting of models.

### Immune cell infiltration analysis

CIBERSORT^18^ is a renowned used method for evaluating immune cell types in tumor microenvironments. Using support vector regression, the expression matrix of immune cell subtypes is deconvoluted. Five hundred forty seven biomarkers distinguish 22 phenotypes of human immune cells, including T-cells, B-cells, plasma cells, and myeloid cells. The CIBERSORT algorithm was used to infer the relative proportions of the 22 immune infiltrating cells in a spearman correlation analysis of gene expression and immune cell content from UM patients.

### Drug sensitivity analysis

The R package ‘pRRophetic’^19^ is employed to forecast the chemotherapy sensitivity of each tumor sample using the largest pharmacogenomics database (GDSC, Genomics Database for Cancer Drug Sensitivity, https://www.cancerrxgene.org/)^20^. To evaluate the validity of regression and prediction, 10 cross-validations were done using the GDSC training set to yield IC50 estimations for each chemotherapeutic drug treatment. Each parameter has been set to the default value, including ‘combats’ to remove batch effects and the averaging of duplicate gene expressions.

### GeneMANIA Analysis

Genemania (http://www.genemania.org)^21^ is a flexible and easy-to-use PPI network building database for visualizing and evaluating functional networks between genes. By using this site, users will be able to set up data sources of gene nodes to be analyzed with various bioinformatics approaches such as physical interaction, gene co-expression, gene co-localization, gene enrichment analysis, and site prediction. Genemania generated a gene network of MMP9/GRIN2A/ACAN to explore its probable mode of action in UM patients.

### Gene set variation analysis (GSVA)

The GSVA package^22^ was used to calculate the expression levels of hub genes in the TCGA datasets. Based on the expression values of the risk score and the hub genes term, a correlation analysis was performed. In this study, *P*<0.05 was deemed statistically significant.

### Cell culture, GRIN2A silencing, and proliferation assay

The human UM cell line C918 and the human retinal pigment epithelium cell line ARPE-19 were cultured in RPMI-1640 medium with 10% FBS and 1% penicillin/streptomycin in an incubator at 37°C under 5% CO_2_, and split when they reached 80% confluence. This study did not involve human or animal subjects, and thus, no ethical approval was required. The study protocol adhered to the guidelines established by the journal.

C918 cells (4×10^4^ cells/ml) were transfected with Lipofectamine 2000 (Invitrogen) plus 50 nM small interfering RNA (siRNA) targeting GRIN2A gene (Small interfering RNA sequence: siRNA1, Sense ‘CGGCAGAAGGAUAACCUCAAU’, Antisense ‘AUUGAGGUUAUCCUUCUGCCG’, siRNA2, Sense ‘CGGAGAGAAACAUUCGGAAUA’, Antisense ‘UAUUCCGAAUGUUUCUCUCCG’) or with the corresponding nonspecific control siRNA (siRNA nonspecific) (Tsingke Biotechnology Co., Ltd.).

To ensure cells adhered to the plates, they were seeded onto 96-well plates (2000 cells/well) and incubated for 12 h. The CCK-8 solution was introduced to the wells at the designated timepoint. Following this, the plate was incubated at 37°C for another 2 h. The viability of the cells was then determined using an ELx808TM Absorbance Microplate Reader (BioTek) to measure their optical absorbance at 450 nm.

### RT-qPCR and Western blot

RNA was isolated from cells treated with different conditions using a total RNA isolation kit and reverse transcription was performed with an Evo M-MLV RTase Enzyme Mix. The transcript levels of GRIN2A were measured using real-time fluorescent quantitative PCR (RT-qPCR) with SYBR Green Pro Taq HS Premix and ABI System Sequence Detector 7500. Primer sequences are listed as follows: GRIN2A (forward, 5′-GTCCTTCTCCGACTGTGAGC-3′; reverse, 5′-TTCCACGATGACGAATGGGG-3′) and GAPDH (forward, 5′-GCACCGTCAAGGCTGAGAAC-3′; reverse, 5′-TGGTGAAGACGCCAGTGGA-3′).

The proteins were extracted with RIPA buffer (Beyotime). An assay kit for measuring protein concentration was used (Beyotime). Proteins were isolated by sodium dodecyl sulfate-polyacrylamide gel electrophoresis (SDS-PAGE). Gels were blotted using the Trans-Blot Turbo Blotting System (BioRad), followed by membrane transfer. Protein Free Rapid Block Buffer (PS108, EpiZyme) was used to block the membrane for 10 min and primary antibodies were incubated against NMDAR2A (1:1000, ab124913, Abcam) and GAPDH (1:1000, #AF7021, Affinity) at 4°C overnight, followed by incubation of antirabbit/mouse Horseradish peroxidase (HRP)-conjugated secondary antibody (1:5000, Promoter) at room temperature for 2 h. High-sensitivity ECL (Beyotime) was used to visualize protein bands. Image J was used to quantify Western blot data.

### Statistical analysis

The statistical analyses were performed using the R language (version 3.6). In all statistical analyses, two-sided tests were used and *P*<0.05 was considered statistically significant.

## Results

### Identification of differentially expressed maps related to chromosome 3 loss

**Figure 1:**
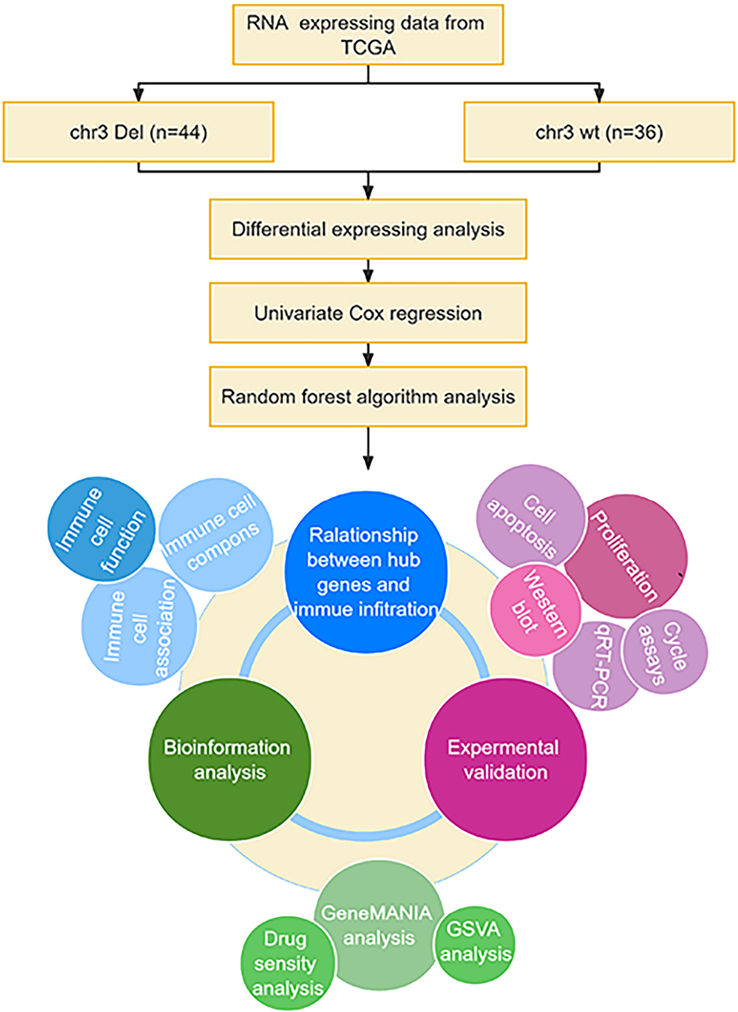
The study flowchart.

**Figure 2:**
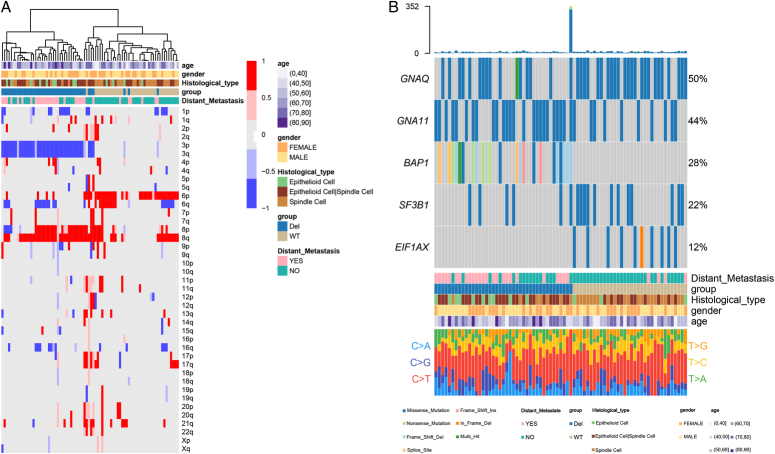
(A) Heatmap shows the distinct genomic copy number profiles in chr3 Del versus chr3 WT group of UM (diploid=0, gray). (B) Significantly mutated genes in chr3 Del versus chr3 WT group of UM.

**Figure 3:**
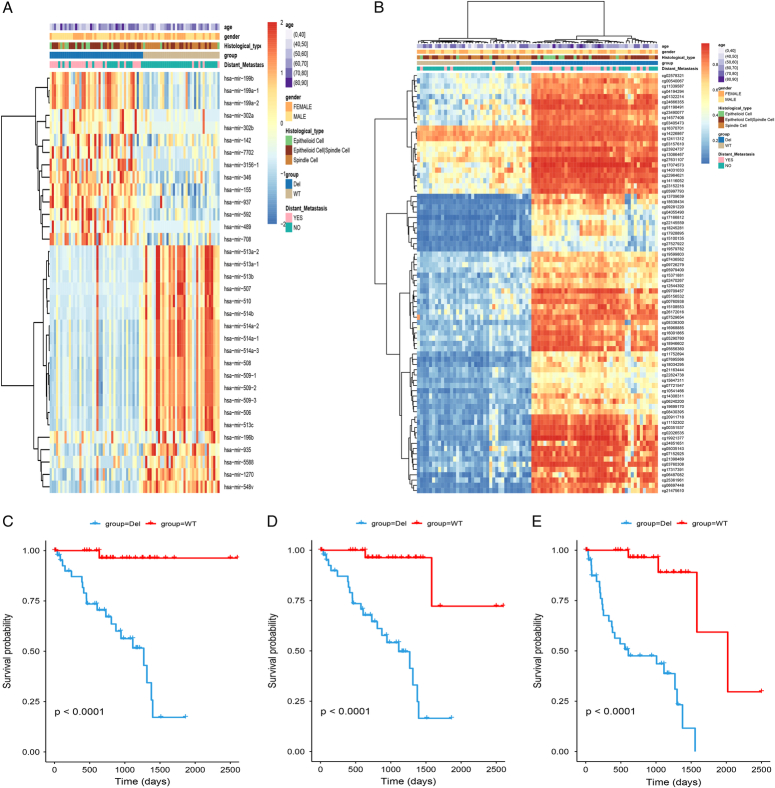
(A) Heatmap shows the distinct RNA-seq data of miRNA expression in chr3 Del versus chr3 WT group of UM. (B) Heatmap shows the distinct RNA-seq data of mRNA expression in chr3 Del versus chr3 WT group of UM. (C) The disease-specific survival (DSS) of chr3 Del and chr3 WT group in UM. (D) The overall survival (OS) of chr3 Del and chr3 WT group in UM. (E) The progression-free interval (PFI) of chr3 Del and chr3 WT group in UM.

The workflow of this research is depicted in Figure 1. To assess the impacts of structural variants on prognosis in UM, we used TCGAbiolinks to download a total of 80 cases with mutation data, expression data, copy number data, and methylation data from the Cancer Genome Atlas Uveal melanoma cohort (TCGA-UM). Copy number variant (CNV) data measured by SNP Array 6.0 were analyzed using GISTIC2 with default parameters to identify the most significant independent regions which were supposed to be driver mutations, and then 80 UM patients were divided into chr3 Del group and chr3 WT group (44 vs 36). As shown in Figure 2A, the chr3 Del group’s patients were more prone to develop distant metastases. We further analyzed the relationship between the chromosome 3 copy number alterations and clinical outcome (UM survival) using the survival R package Kaplan–Meier method and log-rank test (*P*<0.05) the R-package ‘survival’. OS, DSS, and PFI were significantly lower (*P*<0.0001) in the chr3 Del group than in the chr3 WT group (Figs 3C, D, and E). In addition, five significantly mutated genes (SMGs) were detected in 80 UM samples: GNAQ (50%), GNA11(44%), BAP1(28%), SF3B1(22%), and EIF1AX (12%). According to the waterfall plot, BAP1, a tumor suppressor gene located on 3p21.1, often alters in patients with chromosome three copy number loss, resulting in homozygous BAP1 mutations (Fig. 2B). Differential levels of methylation, miRNA, and mRNA expression were found between the two groups of samples (Fig. 3A and B).

### Pathway analyses in UM

**Figure 4:**
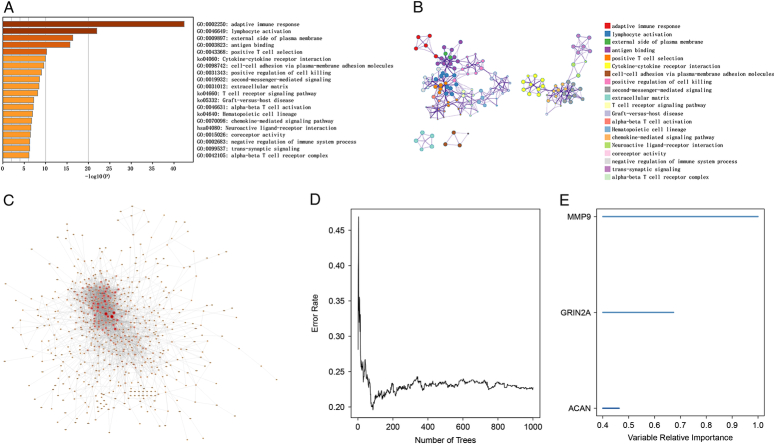
(A) The first 20 enriched terms are shown as a bar char. (B) The network diagram is constructed with each enrichment term as a node and the similarity of the node as the edge. Nodes with the same cluster ID are the same color. (C) PPI network of genes from the green module. The higher the number of connected nodes, the larger the size of the node. (D) Random forest algorithm was used to downsize the different expressed genes (DEGs). (E) The relative importance for three signatures generated by random forest algorithm.

To understand the role of chromosome 3 loss in UM progression, we explored the genetic variations and pathways in the TCGA dataset. The results showed that signatures were mainly enriched in immune-related signaling pathways in UM such as adaptive immune response, lymphocyte activation, external side of plasma membrane, antigen binding, and positive T cell selection (Fig. 4A and B). As part of group efforts to identify the hub genes within different expressed genes (DEGs), gene interaction networks are constructed using the STRING database and visualized them using Cycscape software. As a result, the top 100 genes for association were selected for a subsequent analysis (Fig. 4C).

### Identification of the hub genes in UM

We extracted the top 100 progress related gene expression profiles from the TCGA-UM samples and performed the randomized survival forest algorithm to downsize the candidate lists. After iteration, our study showed that three of these genes were strongly associated with UM prognosis, including MMP9, GRIN2A, and ACAN (Fig. 4D and E). Further investigation of the potential mechanisms of action of these hub genes in UM patients, along with the description of the molecular profile associated with UM, will contribute to the therapeutic management of this disease.

### Association of hub genes expression with immune infiltration

**Figure 5:**
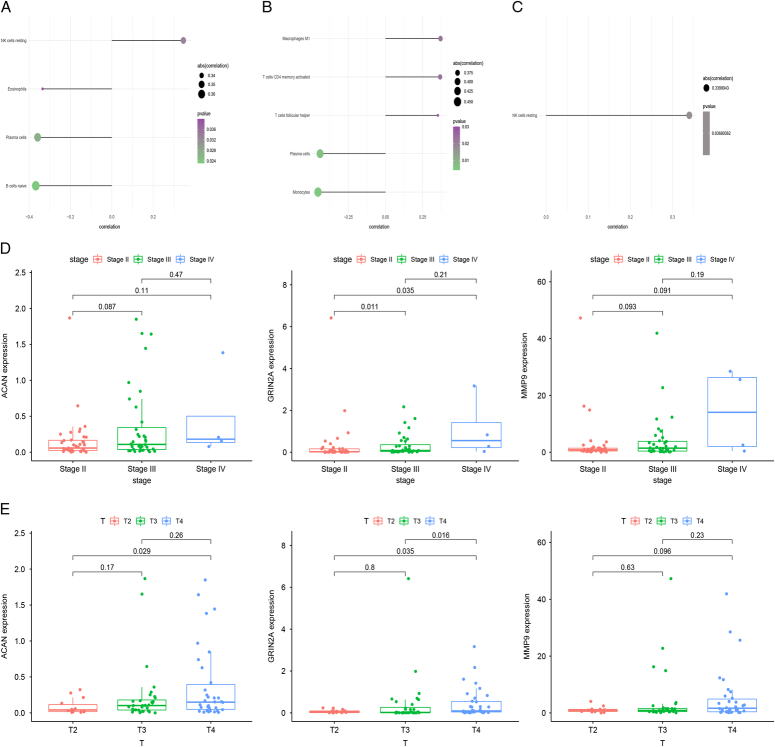
(A) Lollipop plots illustrated the correlation between ACAN expressions and the immune cells. (B) Lollipop plots illustrated the correlation between GRIN2A expressions and the immune cells. (C) Lollipop plots illustrated the correlation between MMP9 expressions and the immune cells. (D) Box plot of the hub genes for different pathological stages. (E) Box plot of the hub genes for different tumor grades.

A tumor microenvironment consists primarily of tumor-associated fibroblasts, immune cells, extracellular matrix, growth factors, inflammatory factors, as well as cancer cells themselves. The microenvironment of a tumor plays a critical role in determining its diagnosis, survival outcome, and sensitivity to clinical treatment. Through the analysis of the relationship between hub genes and tumor immune infiltration in the TCGA dataset, the potential molecular mechanisms are explored by which core genes may influence UM progression. According to the results, all three genes were strongly correlated with immune cell content (Figs 5A, B, and C). Our analysis of the relationship between these three core genes and UM staging revealed that GRIN2A had an effect on UM staging (Fig. 5D and E).

### Association of hub genes expression with drug sensitivity

**Figure 6:**
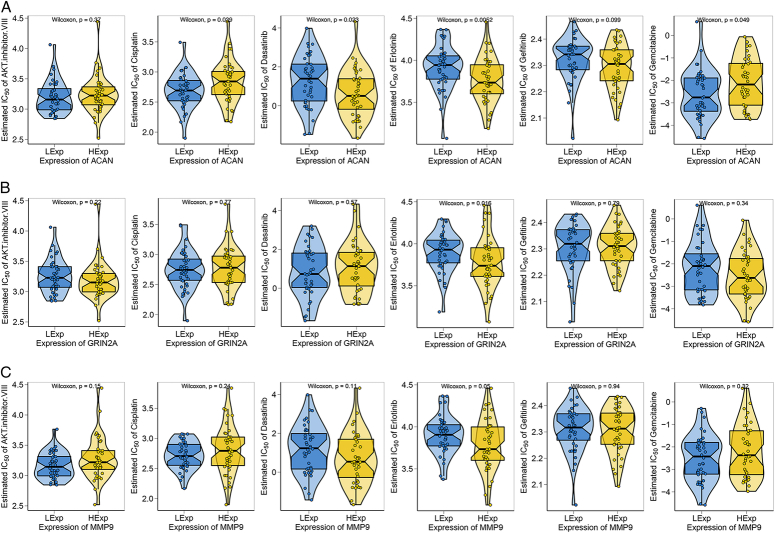
(A) Drug sensitivity analysis of low versus high expression of ACAN group. (B) Drug sensitivity analysis of low versus high expression of GRIN2A group. (C) Drug sensitivity analysis of low versus high expression of MMP9 group.

The GDSC drug sensitivity data and the R package ‘pRRophetic’ were used to further explore the chemosensitivity of core genes to common chemotherapeutic drugs. According to the results, ACAN interfered with Cisplatin, Dasatinib, Erlotinib, and Gemcitabine IC50 values. Erlotinib IC50 values were affected by GRIN2A and MMP9. As a result, it is possible that the core genes will influence patients’ sensitivity to chemotherapy (Fig. 6).

### Construction interaction network of the hub genes

**Figure 7:**
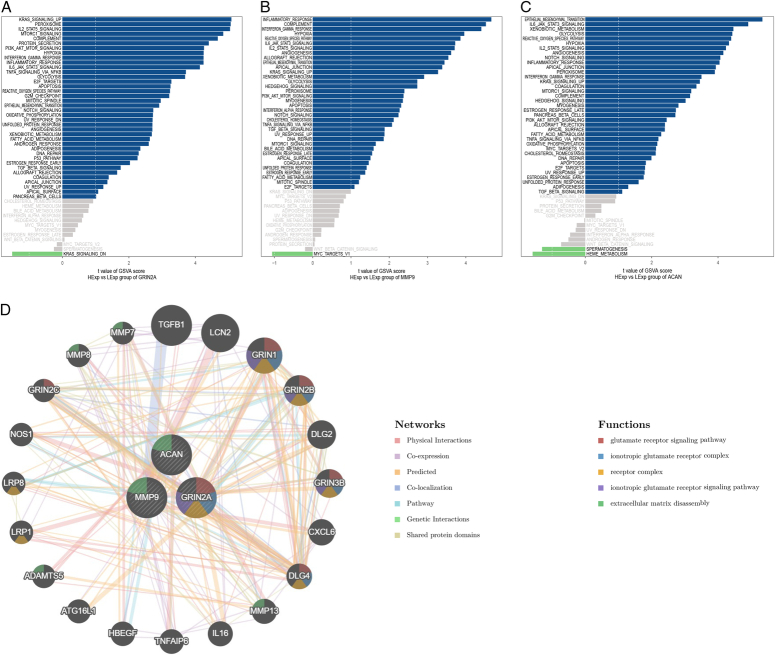
(A) Gene set variation analysis of hub gene’s expression in data retrieved from The Cancer Genome Atlas dataset. (B) The gene–gene interaction network for hub genes was analyzed using the GeneMANIA database. The 20 most frequently changed neighboring genes are shown.

Further investigation into the signaling pathways associated in the hub genes was conducted to explore the potential molecular mechanisms through which the hub genes influence UM progression. GSVA results suggest that high expression of the core genes is associated with FATTY_ACID_METABOLISM, and XENOBIOTIC_METABOLISM, suggesting that these genes may affect disease progression by regulating metabolism (Fig. 7A). In Figure 7B, the interactions between the three core genes are illustrated.

### Experimental validation analysis

**Figure 8:**
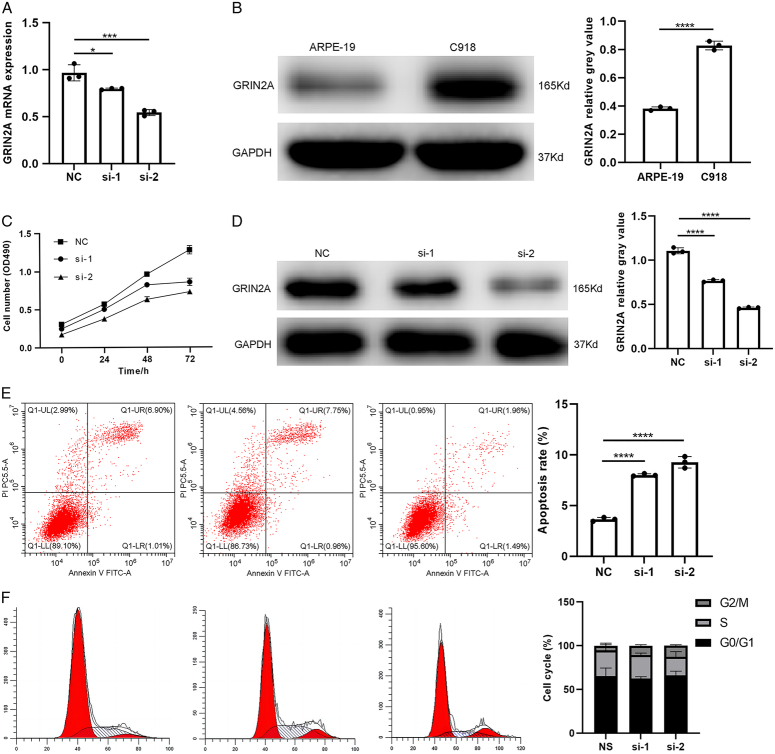
(A) GRIN2A mRNA expression in siRNA treated cells. (B) GRIN2A protein expression in ARPE-19 and C918 cells. (C) CCK-8 assay of C918 cells treated with siRNA. (D) GRIN2A protein expression in siRNA treated cells. (E) Flow cytometric analysis of apoptosis. (F) Flow cytometric analysis of cell phase.

GRIN2A was selected for further biological experiment analysis in an effort to further verify the accuracy and reliability of the bioinformatics analysis. As shown in Figure 8B, Western blot analysis of UM cell line C918 revealed a greater presence of GRIN2A protein than that detected in human normal retinal pigment epithelium cell line ARPE-19. By knocking out GRIN2A with siRNA, the reduction efficiency was confirmed by RT-qPCR and western blotting (Fig. 8A and D). Cell Counting Kit-8 (CCK-8) was used to evaluate C918 cell proliferation ability by lowering expression levels of GRIN2A. Cell line proliferation was reduced when GRIN2A was knocked out (Fig. 8C). Flow cytometry was used to determine the apoptotic rate of GRIN2A in order to assess its biological activity. Si-1 or si-2 transfected groups showed a significant reduction in apoptotic cells after 48 h compared with the control group (Fig. 8E). It was found that GRIN2A significantly impacts the apoptosis of UM. However, GRIN2A knock down has no significant influence on cell phase in C918 cells compared with control group cells (Fig. 8F).

## Discussion

UM patients with chromosome 3 loss are more likely to develop metastases, which is correlated with adverse prognoses^23^. Further, monosomy 3 in UM is associated with immune infiltrates^24^ and poor treatment response^25^, as well as poorer overall survival, compared to those lacking chromosome 3 variants. Thus, chromosome 3 deletion was potentially diagnostic and prognostic for UM. The study’s primary objective was to obtain comprehensive multiomics maps and potential key biomarkers of monosomy 3 in the TCGA-UM cohort, which would assist in exploring the specific molecular networks and identifying the mechanisms underlying UM metastasis.

The TCGA database research network is currently the largest cancer gene information database, storing data including gene expression data, miRNA expression data, copy number variation data, DNA methylation data, and SNP data, etc. Correspondingly, TCGAbiolinks were utilized to download and then perform integrative analyses between two groups of TCGA data. SNP-related data of UM has been processed along and the raw mRNA expression data was downloaded, as the raw SNP data in TCGA are not available to the public. UM patients are divided into two cohorts, samples with chromosome 3 loss (chr3 Del group) and samples with normal copy numbers of chromosome 3 (chr3 WT group), each with an identical admixture of histology-stage combinations. Later trials revealed that the results differed considerably between the two groups, as previously reported. The extremely divergent results reported by the two groups indicate that chr3 plays a major role in the process. The most frequent mutations identified in our study were GNAQ, GNA11, BAP1, SF3B1, and EIF1AX mutations, and we find that BAP1 of these five mutations was present and could clearly differentiate chr3 del group patients from chr3 wt group samples. Analyzing these genes for the different presence of mutation between two groups according to chromosome 3 status may suffer for diagnostic purposes.

It has become increasingly necessary to apply deep learning on large-scale multi-omics datasets of UM from open sources such as TCGA, CCLE4, and GDSC5 in order to broaden our understanding of UM molecular events. According to Robertson *et al*.^8^, a comprehensive multiplatform analysis identified four molecularly distinct subtypes, which are clinically relevant. Vichitvejpaisal *et al*.^26^ used the TCGA classification for the prognostication of UM using a large cohort study. Xue *et al*.^27^ developed an 18-gene signature based on univariate Cox regression analysis and found that high-risk individuals had more chromosome aberrations than low-risk individuals. Liu *et al*.^28^ constructed a 6-gene signature with prognostic values related to glycolysis and immune response.

A machine learning-based predictive algorithm may be used to unravel the intricate workings of systems biology using data from various omics sources^29^. With the development of machine learning methods, it is now feasible to integrate and evaluate various omics data allowing for the discovery of novel biomarkers, that can assist in disease prediction, patients stratification, and the delivery of precision medicine^30^. In machine learning and data mining, random survival forests (RSF) extend random forest to right-censored survival and competing risk settings. Several clinical studies have employed this strategy effectively, and in some cases, it has been shown to outperform classical statistical methods^17^. Sun *et al*.^31^ constructed a tumor-infiltrating CD8+ T-cell gene signature using multiple machine learning algorithms. To predict the risk of metastasis, Vaquero-Garcia *et al*.^32^ used machine learning methods, including logistic regression, decision trees, survival random forests, and survival-based regression models. The research above provides information about new candidate genes, a molecular landscape of UM, and novel molecular classifications that describe molecular tumor heterogeneity. This information is relevant to new targeted cancer therapies.

Previous whole-exome sequencing studies have shown melanoma tumors with GRIN2A mutations. The GRIN2A gene encodes the regulatory GluN2A subunit of the glutamate-gated N-methyl-d-aspartate receptor (NMDAR), and its involvement in melanoma remains unclear. Interestingly, in vitro research revealed that GRIN2A is overexpressed in UM cells and plays an important role in the proliferation in UM cell proliferation.

In short, this study represented a comprehensive assessment of the molecular alterations association with chromosome 3 loss in the TCGA-UM cohort and proposed that GRIN2A could be useful in diagnosing, treating, and prognosing patients with disease. Animal models and further experiment validation are required.

## Ethical approval

This study did not involve human or animal subjects, and thus, no ethical approval was required. The study protocol adhered to the guidelines established by the journal.

## Consent

This study was prepared using the open-access dataset of a published paper. Therefore, patient consent is not required for this study.

## Sources of funding

This research did not receive any specific grant from funding agencies in the public, commercial, or not-for profit sectors.

## Author contribution

X.Y.: data collection and writing; T.K.: data collection and formal analysis; T.L.: data collection and writing; S.L.: methodology and data collection; X.H.: conceptualization and writing; X.H.: formal analysis and data collection; X.Y.: methodology; F.Z.: review; J.Z.: investigation; Q.Y.: project administration.

## Conflicts of interest disclosure

The authors declare that they have no known competing financial interests or personal relationships that could have appeared to influence the work reported in this paper.

## Research registration unique identifying number (UIN)

1. Name of the registry: not applicable.
2. Unique identifying number or registration ID: not applicable.
3. Hyperlink to your specific registration (must be publicly accessible and will be checked): not applicable.

## Guarantor

Xi Yong.

## Data availability statement

Publicly available.

## Provenance and peer review

Not commissioned, externally peer-reviewed.
